# Geriatric nutritional risk index predicts prognosis in hepatocellular carcinoma after hepatectomy: a propensity score matching analysis

**DOI:** 10.1038/s41598-021-88254-z

**Published:** 2021-04-27

**Authors:** Hiroki Kanno, Yuichi Goto, Shin Sasaki, Shogo Fukutomi, Toru Hisaka, Fumihiko Fujita, Yoshito Akagi, Koji Okuda

**Affiliations:** Department of Surgery, Kurume University School of Medicine, 67 Asahi-machi, Kurume, Japan

**Keywords:** Cancer, Gastroenterology

## Abstract

The geriatric nutritional risk index (GNRI) is widely used for nutritional assessment in older inpatients and is associated with postoperative complications and cancer prognosis. We investigated the use of GNRI to predict long-term outcomes in hepatocellular carcinoma of all etiologies after hepatectomy. Overall, 346 patients were examined after propensity score matching. We dichotomized the GNRI score into high GNRI (> 98: N = 173) and low GNRI (≤ 98: N = 173) and evaluated recurrence-free survival (RFS) and overall survival (OS) between both groups. Clinicopathological characteristics between the low- and high-GNRI groups were similar after propensity score matching except for the components of the GNRI score (body mass index and serum albumin level), Child–Pugh score (comprising serum albumin level), and preoperative alpha-fetoprotein level (*p* < 0.0001, *p* < 0.0001, *p* = 0.0030, and *p* = 0.0007, respectively). High GNRI was associated with significantly better RFS and OS (*p* = 0.0003 and *p* = 0.0211, respectively; log-rank test). Multivariate analysis revealed that GNRI is an independent prognostic factor of RFS and OS (low vs. high; hazard ratio [HR], 1.8284; 95% confidence interval [CI] 1.3598–2.4586; *p* < 0.0001, and HR, 1.5452; 95% CI 1.0345–2.3079; *p* = 0.0335, respectively). GNRI is an objective, inexpensive, and easily calculated assessment tool for nutritional status and can predict prognosis of hepatocellular carcinoma after hepatectomy.

## Introduction

Hepatocellular carcinoma (HCC) is one of the leading causes of cancer-related deaths worldwide^1^. Despite improvements in therapeutic options such as surgery, ablation, transarterial chemoembolization, and molecular targeted agents, the outcome of HCC is unsatisfactory owing to the high recurrence rate. Chronic hepatitis or liver cirrhosis is usually present in the background of HCC and may result in malnutrition in these patients. Some studies have reported that malnutrition negatively impacts postoperative complications and cancer prognosis^2^.

Inflammation, malnutrition, and cancer have a close relationship with each other. Malnutrition causes the deterioration of tumor immunity, leading to postoperative complications and cancer progression^3,4^. Furthermore, cancer-related systemic inflammation induces catabolism of patients, which causes metastasis^5,6^. Some inflammatory indices—neutrophil/lymphocyte ratio, platelet/lymphocyte ratio, and the modified Glasgow prognostic score—and several nutritional indices—the prognostic nutritional index, controlling nutritional status score (CONUT), sarcopenia, and geriatric nutritional index (GNRI)—can predict postoperative complications and long-term outcomes of several cancers^7–9^. These scores are also useful for evaluating postoperative complications and prognosis of HCC^10–12^.

The GNRI was proposed by Bouillanne et al. in 2005^13^ and has been widely used for nutritional assessment in older inpatients with cardiovascular diseases, chronic renal failure with hemodialysis, and chronic liver disease^14–17^. The GNRI is calculated using serum albumin concentration, body height, and body weight, and patients are classified into four nutritional risk grades. Recently, some researchers have reported that the GNRI is associated with postoperative complications and cancer prognosis^18,19^. However, only one report has described this relationship in HCC, and this study was limited to patients aged 65 years or older who were positive for the hepatitis B virus^20^. However, the median age of onset of HCC is around 65 years old, and hepatitis virus C is the major cause of HCC in the developed countries. Therefore, we investigated the use of the GNRI to predict long-term outcomes in HCC patients, including those younger than 65 years of age with all underlying liver diseases.

## Materials and methods

### Patients

Six hundred and forty-eight consecutive patients who underwent hepatectomy for initial HCC at Kurume University between 2008 and 2018 were retrospectively reviewed. Of these, 120 were excluded due to the following reasons: 107 underwent preoperative treatment such as transcatheter arterial infusion, transarterial chemoembolization, and/or ablation; six did not have histologically confirmed HCC (HCC + combined hepatocellular-cholangiocellular carcinoma, necrosis, etc.); three had insufficient data; two did not achieve curative resection; one had another synchronous malignancy; and one had inferior vena cava tumor thrombus. Therefore, 528 patients were finally enrolled for analyses before propensity score matching (PSM). This study was approved by the Research Ethics Committee of Kurume University (No. 20236) and was conducted according to the tenets of the Declaration of Helsinki. The need for informed consent was waived owing to the retrospective nature of the study.

### Data collection and calculation of the GNRI scores

Clinical and pathological data were collected from the patients’ medical records. Blood samples and physical data were obtained within 1 week before surgery. Calculation of the GNRI score was as follow: GNRI = (1.489 × serum albumin concentration [g/L]) + (41.7 × present/ideal body weight [kg]). When the present body weight exceeded the ideal body weight, “present/ideal body weight” was set to 1. Pathological diagnosis was performed by two pathologists in accordance with the rules of the Liver Cancer Study Group of Japan.

### Cutoff value of the GNRI score

The GNRI was categorized into four risk groups: no risk (GNRI: > 98), low risk (GNRI: 92 to 98), moderate risk (GNRI: 82 to < 92), and major risk (GNRI: < 82). We set the cutoff value to ≤ 98 or > 98, which was in line with other studies^18,21^. The distribution of the GNRI score in the present study is shown in Supplementary Fig. S1.

### Surgical procedure

The type of surgery was comprehensively planned according to the Child–Pugh score, liver damage, platelet count, liver stiffness, extent of cirrhosis, presence of esophageal varix and splenomegaly, tumor number, tumor location, and patients’ comorbidities. If poor liver function with Child–Pugh score C, elevated bilirubin level, or low platelet count was observed and/or portal hypertension was suspected before or during operation, we did not perform the surgery. Major hepatectomy was defined as lobectomy or more, and minor hepatectomy was defined as sectionectomy or less. Surgical procedures were performed as follows. Liver mobilization was performed in most cases, if necessary. The Pringle maneuver was used in most cases according to the surgeon’s preference. Parenchymal transection was carried out using an ultrasonic coagulation dissector and/or clamp crushing methods. Sizable vasculature was ligated tightly.

### Postoperative follow-up

In the postoperative follow-up, patients were examined using ultrasonography and tumor markers (alpha-fetoprotein [AFP] and/or protein induced by vitamin K absence or antagonist-II [PIVKA-II]) at least every 3 months after surgery. Additionally, computed tomography (CT) and/or magnetic resonance imaging (MRI) were performed at least every 6 months in the patients with a high risk of recurrence. If recurrence was suspected, further examinations such as gadolinium ethoxybenzyl diethylenetriamine pentaacetic acid-enhanced MRI or contrast-enhanced ultrasonography were conducted. Recurrence-free survival (RFS) was defined as the time from surgery to recurrence or death. Overall survival (OS) was defined as the time from surgery to death.

### Statistical analyses

The clinicopathological characteristics were compared using the chi-square test for categorical variables and the Mann–Whitney U test for continuous variables. PSM was performed to overcome selection bias. The propensity score was calculated using a logistic regression model. Covariates entered into the PSM model were as follow: age, sex, underlying liver disease, total bilirubin level, prothrombin time, platelet count, AFP level, duration of surgery, estimated blood loss, surgical procedure, tumor diameter, tumor number, tumor differentiation, microvascular invasion (MVI), and histological fibrosis grade. PSM was performed using a 1:1 matching method with a caliper width of 0.2, and the area under the curve calculated from the receiver operating characteristics curve was 0.71734 (*p* < 0.0001). Survival curves were created using the Kaplan–Meier method and compared using the log-rank test. A Cox proportional hazards model was used for univariate and multivariate analyses, and hazard ratios (HRs) and 95% confidence intervals (CIs) were calculated. All statistical analyses were performed using JMP Pro, version 15 (SAS Institute, Cary, NC). A *p* value < 0.05 was considered statistically significant.

### Ethics approval

This study was approved by the Research Ethics Committee of Kurume University (Approval number: No. 20236) and was conducted in accordance with the Declaration of Helsinki. The need for informed consent was waived owing to the retrospective nature of the study.

## Results

### Patient characteristics

Clinicopathological features between the low- and high-GNRI groups in the entire cohort before PSM are summarized in Supplementary Table S1. Age, body mass index (BMI), underlying liver disease, total bilirubin level, serum albumin concentrations, prothrombin time, AFP, Child–Pugh score, duration of surgery, and tumor number were significantly different between the two groups (*p* = 0.0002, *p* < 0.0001, *p* < 0.0001, *p* = 0.0150, *p* < 0.0001, *p* = 0.0005, *p* = 0.0001, *p* = 0.0004, *p* = 0.0126, and *p* = 0.0286, respectively). After matching, almost all variables were adjusted except for the components of the GNRI score (BMI and serum albumin level), Child–Pugh score (consisting serum albumin level), and preoperative AFP level (*p* < 0.0001, *p* < 0.0001, *p* = 0.0030, and *p* = 0.0007, respectively) (Table 1).

**Table 1:** Clinicopathological characteristics of the patients in the low- and high-GNRI groups after propensity score matching.

Characteristics (n = 346)	GNRI low (n = 173)	%	GNRI high (n = 173)	%	Total	*P* value
Age (years), median (range)	73 (43–87)		72 (33–89)			0.4644
Sex						0.4163
Male	125	72.3	128	74.0	253	
Female	48	27.7	45	26.0	93	
BMI, median (IQR)	21.7 (19.3–24.45)		23.5 (21.75–25.65)			< 0.0001
Underlying liver disease						0.7165
HBV	14	8.1	9	5.2	23	
HCV	108	62.4	112	64.7	220	
B + C	4	2.3	3	1.7	7	
NonBnonC	47	27.2	49	28.3	96	
Excessive alcohol consumption*						0.2486
Yes	35	20.2	44	25.4	79	
No	138	79.8	129	74.6	267	
DM						0.3641
Yes	55	31.8	63	36.4	118	
No	118	68.2	110	63.6	228	
T.bil (mg/dL), median (IQR)	0.71 (0.57–0.85)		0.73 (0.59–0.90)			0.3933
Albumin (g/dL), median (IQR)	3.63 (3.41–3.77)		4.18 (3.98–4.38)			< 0.0001
PT (%), median (IQR)	91 (80.5–100)		91 (82.0–100)			0.5820
Platelet (× 10^4^/L), median (IQR)	13.7 (10.2–17.9)		14.9 (11.5–18.2)			0.1466
AFP (ng/mL), median (IQR)	16.9 (5.4–101.25)		6.6 (3.8–33.7)			0.0007
Child–Pugh						0.0030
A	163	94.2	172	99.4	335	
B	10	5.8	1	0.6	11	
Surgical procedure						
Major	54	31.2	57	32.9	111	0.7297
Minor	119	68.8	116	67.1	235	
Duration of surgery (min), median (IQR)	354 (289–434)		350 (273–449)			0.7790
Estimated blood loss (ml), median (IQR)	385 (175–678)		330 (78–818)			0.1785
Tumor diameter (mm), median (IQR)	27 (19.5–39)		26 (20–40)			0.9118
Tumor number						0.6896
Solitary	139	80.3	136	78.6	275	
Multiple	34	19.7	37	21.4	71	
Differentiation						0.6680
Well/mod	142	82.1	145	83.8	287	
Poor	31	17.9	28	16.2	59	
Microvascular invasion						1.0000
Yes	93	53.8	93	53.8	186	
No	80	46.2	80	46.2	160	
Histological fibrosis grade						0.7466
F0-2	80	46.2	83	48.0	163	
F3-4	93	53.8	90	52.0	183	
GNRI, median (IQR)	94.0 (90.2–96.4)		102.7 (100.2–105.7)			< 0.0001

*Excessive alcohol consumption is defined as > 28 g/day ethanol in men and > 14 g/day in women.

AFP: alpha-fetoprotein, BMI: body mass index, DM: diabetes mellitus, GNRI: geriatric nutritional risk index, IQR: interquartile range, PT: prothrombin time, T.bil: total bilirubin.

### Comparison of survival between the low- and high-GNRI groups

**Figure 1:**
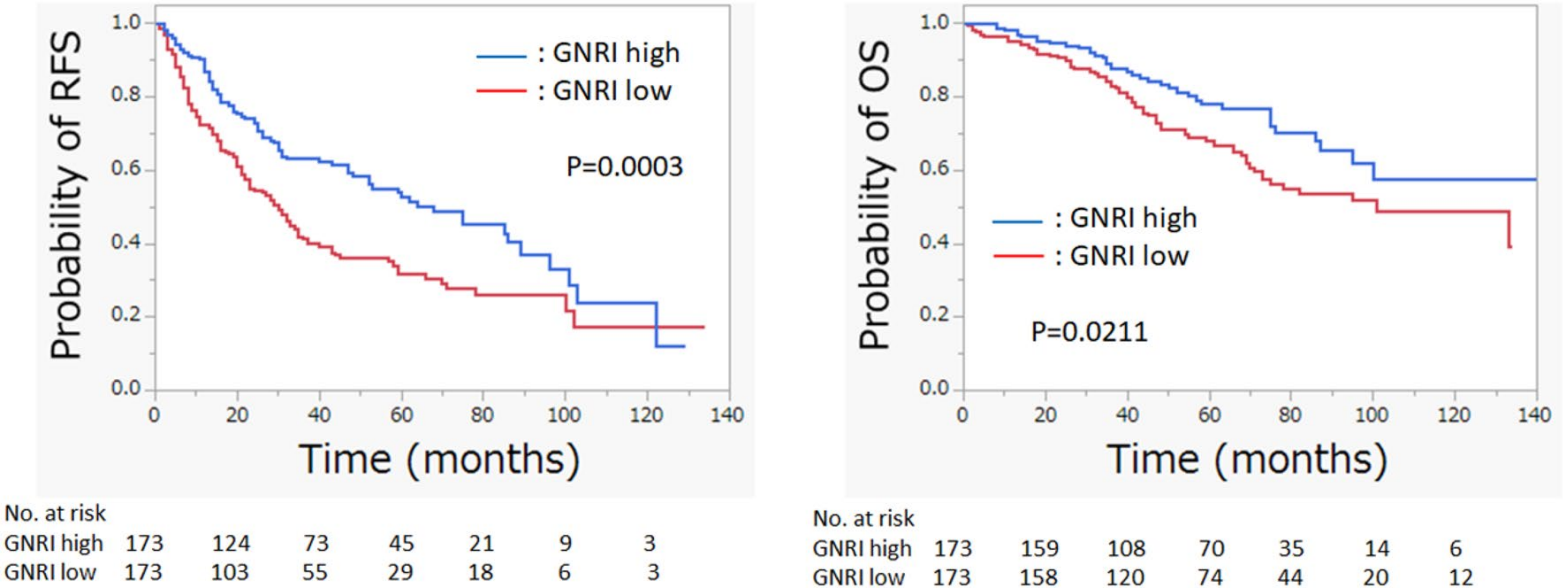
Kaplan–Meier curves of RFS and OS in the low- and high-GNRI groups. RFS, recurrence-free survival; OS, overall survival, GNRI, geriatric nutritional risk index.

The RFS and OS curves of the two groups are shown in Fig. 1. A high GNRI was associated with significantly better RFS and OS (*p* = 0.0003 and *p* = 0.0211, respectively).

### Univariate and multivariate analyses for RFS and OS

The results of the univariate and multivariate analyses of RFS and OS are shown in Tables 2 and 3. Univariate analysis of RFS demonstrated that underlying liver disease (Viral vs. Non-viral; HR, 1.4269; 95% CI 1.0195–1.9970; *p* = 0.0325), AFP level (5 > vs. ≥ 5 ng/mL; HR, 0.5440; 95% CI 0.3864–0.7657; *p* = 0.0005), tumor number (solitary vs. multiple; HR, 0.5588; 95% CI 0.4053–0.7706; *p* = 0.0004), tumor differentiation (well/mod vs. poor; HR, 0.6494; 95% CI 0.4535–0.9298; *p* = 0.0184), MVI (− vs. + ; HR, 0.5099; 95% CI 0.3809–0.6825; *p* < 0.0001), histological fibrosis grade (F0-2 vs. F3-4; HR, 0.6963; 95% CI 0.5229–0.9272; *p* = 0.0133), and GNRI (low vs. high; HR, 1.6809; 95% CI 1.2645–2.2346; *p* = 0.0003) were the prognostic factors. On multivariate analysis, underlying liver disease (Viral vs. Non-viral; HR, 1.4519; 95% CI 1.0265–2.0535; *p* = 0.0351), tumor number (solitary vs. multiple; HR, 0.5645; 95% CI 0.4033–0.7903; *p* = 0.0009), MVI (− vs. + ; HR, 0.4957; 95% CI 0.3637–0.6757; *p* < 0.0001), and GNRI (low vs. high; HR, 1.8284; 95% CI 1.3598–2.4586; *p* < 0.0001) were the independent prognostic factors. Univariate analysis of OS showed that tumor differentiation (well/mod vs. poor; HR, 0.5099; 95% CI 0.3227–0.8059; *p* = 0.0039), MVI (− vs. + ; HR, 0.4837; 95% CI 0.3206–0.7300; *p* = 0.0005), and GNRI (low vs. high; HR, 1.5935; 95% CI 1.0674–2.3789; *p* = 0.0227) were the prognostic factors. On multivariate analysis, MVI (− vs. + ; HR, 0.5464; 95% CI 0.3553–0.8403; *p* = 0.0059), and GNRI (low vs. high; HR, 1.5452; 95% CI 1.0345–2.3079; *p* = 0.0335) were the independent prognostic factors.

**Table 2:** Univariate and multivariate analyses of recurrence-free survival.

RFS	Univariate analysis			Multivariate analysis		
	OR	95% CI	*P* value	OR	95% CI	*P* value
Age (75 > vs. ≥ 75)	1.0433	0.7797–1.3959	0.7752			
Sex (Male vs. Female)	1.2893	0.9260–1.7951	0.1241			
Viral vs. Non-viral	1.4269	1.0195–1.9970	0.0325	1.4519	1.0265–2.0535	0.0351
DM (− vs. +)	0.9823	0.7308–1.3205	0.9060			
AFP (5 > vs. ≥ 5)	0.5440	0.3864–0.7657	0.0005	0.7886	0.5486–1.1337	0.1998
Surgical procedure (Minor vs. Major)	0.9406	0.6971–1.2690	0.6884			
Duration of surgery (300 > vs. ≥ 300)	0.8277	0.6246–1.0968	0.1880			
Estimated blood loss (500 > vs. ≥ 500)	0.8957	0.6719–1.1941	0.4529			
Tumor diameter (20 ≥ vs. > 20)	0.7635	0.5628–1.0358	0.0829			
Tumor number (Solitary vs. Multiple)	0.5588	0.4053–0.7706	0.0004	0.5645	0.4033–0.7903	0.0009
Differentiation (well/mod vs. poor)	0.6494	0.4535–0.9298	0.0184	0.8895	0.6070–1.3035	0.5483
Microvascular invasion (− vs. +)	0.5099	0.3809–0.6825	< 0.0001	0.4957	0.3637–0.6757	< 0.0001
Histological fibrosis grade (F0-2 vs. F3-4)	0.6963	0.5229–0.9272	0.0133	0.7918	0.5879–1.0665	0.1245
GNRI (98 > vs. ≥ 98)	1.6809	1.2645–2.2346	0.0003	1.8284	1.3598–2.4586	< 0.0001

AFP: alpha-fetoprotein, CI: confidence interval, DM: diabetes mellitus, GNRI: geriatric nutritional risk index, OR: odds ratio, RFS: recurrence-free survival.

**Table 3:** Univariate and multivariate analyses of overall survival.

OS	Univariate analysis			Multivariate analysis		
	OR	95% CI	*P* value	OR	95% CI	*P* value
Age (75 > vs. ≥ 75)	0.7049	0.4785–1.0384	0.0769			
Sex (Male vs. Female)	0.9949	0.6410–1.5442	0.9818			
Viral vs. Non-viral	0.9748	0.6244–1.5219	0.9106			
DM (− vs. +)	1.0914	0.7212–1.6516	0.6791			
AFP (5 > vs. ≥ 5)	0.6767	0.4255–1.0763	0.0990			
Surgical procedure (Minor vs. Major)	0.7704	0.5173–1.1473	0.1992			
Duration of surgery (300 > vs. ≥ 300)	0.8989	0.6096–1.3255	0.5907			
Estimated blood loss (500 > vs. ≥ 500)	0.7651	0.5190–1.1279	0.1763			
Tumor diameter (20 ≥ vs. > 20)	0.7332	0.4772–1.1267	0.1569			
Tumor number (Solitary vs. Multiple)	0.7841	0.4996–1.2306	0.2902			
Differentiation (well/mod vs. poor)	0.5099	0.3227–0.8059	0.0039	0.6449	0.3996–1.0407	0.0724
Microvascular invasion (− vs. +)	0.4837	0.3206–0.7300	0.0005	0.5464	0.3553–0.8403	0.0059
Histological fibrosis grade (F0-2 vs. F3-4)	0.7846	0.5309–1.1596	0.2235			
GNRI (98 > vs. ≥ 98)	1.5935	1.0674–2.3789	0.0227	1.5452	1.0345–2.3079	0.0335

AFP: alpha-fetoprotein, CI: confidence interval, DM: diabetes mellitus, GNRI: geriatric nutritional risk index, OR: odds ratio, OS: overall survival.

## Discussion

In the present study, we evaluated the relationship between the GNRI score and the long-term outcomes of HCC after hepatectomy. In the entire cohort, the clinicopathological characteristics were different between the two groups; therefore, PSM was applied to overcome that bias. After PSM, the high-GNRI group demonstrated significantly better RFS and OS according to the log-rank test, and a high GNRI was an independent prognostic factor of better RFS and OS in multivariate analyses. Thus far, only one study has mentioned the relationship between GNRI and the prognosis of HCC. Li et al. reported that a low GNRI resulted in a worse prognosis in HCC; however, their study was limited to hepatitis B virus-associated HCC and patients aged 65 years or older^20^. To the best of our knowledge, this is the first study that evaluated the relationship between GNRI and the long-term outcomes of HCC to include patients younger than 65 years and whole underlying liver disease in HCC.

Some researchers have demonstrated that immune nutritional score could predict postoperative complications^10,18,20,22^. Li et al. reported that preoperative low GNRI score in the HCC cohort was associated with postoperative liver failure grade A and B (defined by the International Study Group of Liver Surgery) and severe complications. Additionally, Li et al. revealed that an early postoperative low CONUT score was associated with Clavien-Dindo grade III-V complications after hepatectomy. Preoperative risk assessment will become increasingly important owing to an increasingly aging population globally.

Many studies have described the usefulness of sarcopenia with respect to postoperative complications and cancer prognosis^23,24^. Although sarcopenia is a powerful tool to evaluate nutritional status, the measurements are complex. Measurements of calf circumference, 6-m walking time, handgrip strength, 5-times sit-to-standing time, and/or the area of the iliopsoas muscle at the third lumbar vertebra level on CT, etc., are required. Child–Pugh score is also a very useful prognostic assessment tool irrespective of surgery^25^. Vitale et al. reported that in Child–Pugh score B patients, survival after surgery was similar with that after locoregional therapies^26^. However, in some institutes, including our own, non-surgical therapies such as ablation and transarterial chemoembolization are preferred in cases with Child–Pugh score B. Indeed, the rate of Child–Pugh score B cases was only 3% in the present study. Therefore, we believe another risk assessment tool is warranted. The GNRI is an objective, inexpensive, and readily available assessment tool of nutritional status. Only serum albumin level, body height, and weight are needed to calculate the GNRI. Shoji et al. reported a correlation between the area of the iliopsoas muscle at the third lumbar vertebra level and the GNRI score^27^. Besides, Cereda and Vanotti indicated that the GNRI was associated with mid-upper arm muscle circumference, arm muscle area, handgrip strength, and handgrip strength/arm muscle area^28^. Therefore, the GNRI may be a useful tool for predicting postoperative complications and prognosis, especially in patients in whom evaluating sarcopenia is challenging and in institutes that lack the appropriate measuring equipment.

Serum albumin concentration is the main screening tool for immune nutritional status. Hypoalbuminemia induces an impaired immune response, and immunity has a strong influence on cancer prognosis^29,30^. Additionally, a low albumin level is associated with elevated inflammatory cytokines such as tumor necrosis factor-alpha, interleukin-1, and interleukin-6, which may lead to the progression of HCC^31,32^. Therefore, a low GNRI may reflect impaired tumor immunity which may cause cancer progression.

BMI is also an immune nutritional index, and several recent studies have mentioned an association between BMI and the response to immune checkpoint inhibitors^33,34^. A high BMI was reported to be associated with improved survival in patients treated with immune checkpoint inhibitors. Cortellini et al. reported that adipose tissue could activate cytotoxic T-cells and decrease regulatory T-cells. Thus, BMI might influence host immunity. The GNRI is based on serum albumin concentration and BMI, and high values for these components can positively influence host immunity which may improve the cancer prognosis.

Our results indicated that preoperative malnutrition causes worse outcomes after hepatectomy in HCC. Interventions aimed at preoperative nutritional status will improve not only short-term outcomes but also cancer prognosis. Several studies have demonstrated that perioperative nutritional support improves morbidity and prognosis^35–37^. Therefore, we believe that perioperative immune nutritional support should be performed intensively for patients who are planned to undergo hepatectomy.

The present study has some study limitations. First, there is a potential risk of selection bias owing to the single-center, retrospective design. Prospective, multi-institutional studies are needed to validate our results. Second, postoperative complications were not assessed in the present study. Third, differences in patient characteristics between the two groups were not completely overcome. Child–Pugh score and AFP level were significantly different between the two groups even after PSM, which could influence prognosis. Additionally, the follow-up protocol was not standardized, which could make RFS data less powerful. Finally, the evaluation of resectability and the preselection of suitable candidates for surgery are quite complex. Even if liver function is not well preserved, surgery is sometimes performed in cases where the tumor is near the liver surface. In contrast, if the tumor is located around the hepatic hilum or the root of the hepatic veins, we sometimes hesitate to perform surgery even in cases with preserved liver function.

In conclusion, the GNRI is an objective, inexpensive, and easily calculated assessment tool for nutritional status. Our findings suggest that GNRI can be a useful predictor of survival in HCC after hepatectomy. Perioperative nutritional support might improve cancer survival.

## Supplementary Information

Supplementary Information
