# Supplementary material for: Geriatric nutritional risk index predicts prognosis in hepatocellular carcinoma after hepatectomy: a propensity score matching analysis
Source: Sci Rep. 2021 Apr 27;11:9038. doi: 10.1038/s41598-021-88254-z (PMC8079680; doi:10.1038/s41598-021-88254-z)
Supplement: Supplementary file 1 — Supplementary Information [file 41598_2021_88254_MOESM1_ESM.docx]

**Geriatric nutritional risk index predicts prognosis in hepatocellular carcinoma after hepatectomy: a propensity score matching analysis**

Hiroki Kanno^*^, Yuichi Goto, Shin Sasaki, Shogo Fukutomi, Toru Hisaka, Fumihiko Fujita, Yoshito Akagi, and Koji Okuda

Department of Surgery, Kurume University School of Medicine, Kurume, Japan

^*^Corresponding author: Hiroki Kanno

Department of Surgery, Kurume University School of Medicine, 67 Asahi-machi, Kurume, Japan

Phone: 819-4231-7902

Fax: 819-4231-7820

E-mail: kanno_hiroki@med.kurume-u.ac.jp

**Supplementary materials**

**Supplementary Figure S1.** Distribution of the GNRI score

**
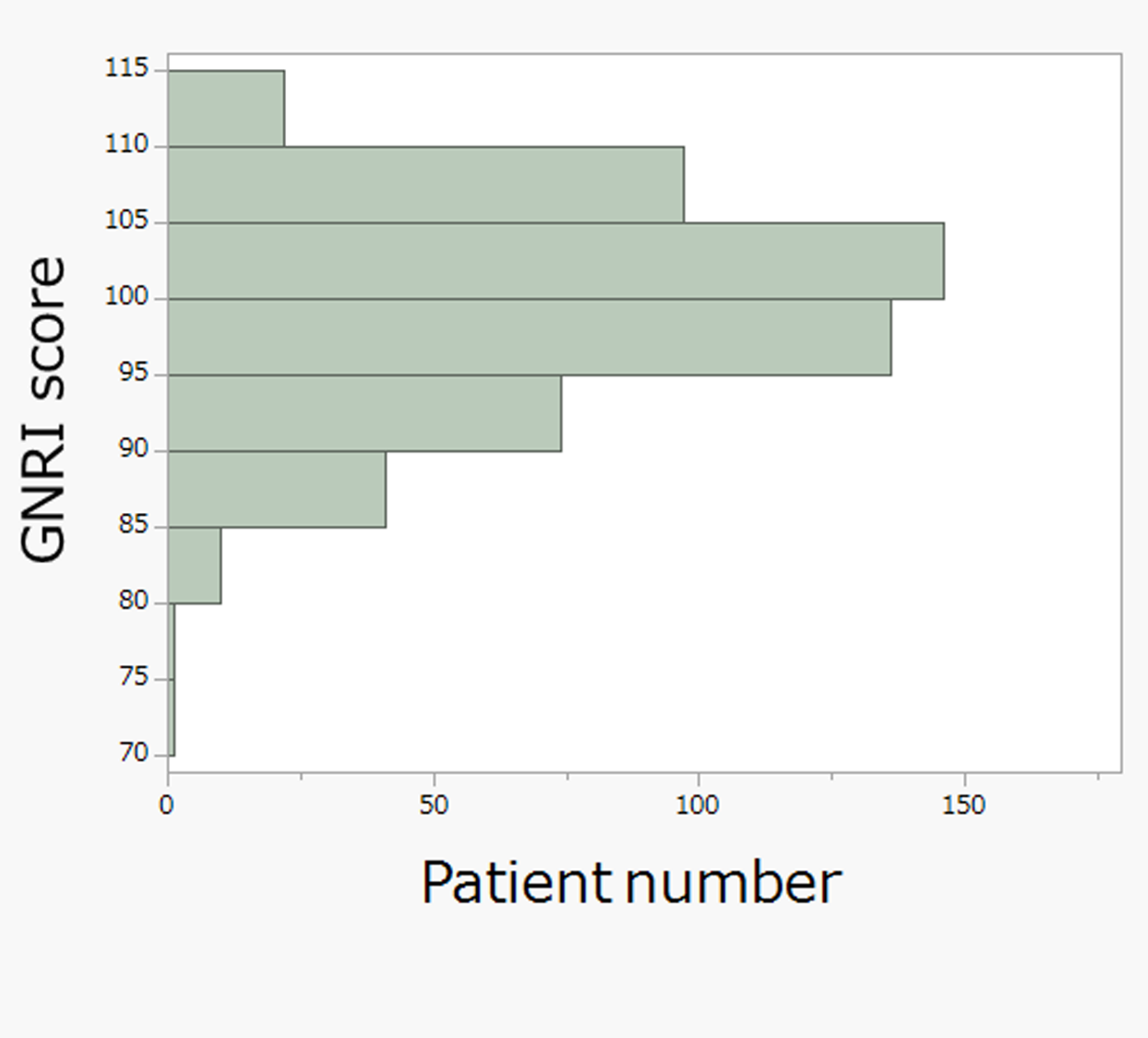
Supplementary Table S1.** Clinicopathological features between the low- and high-GNRI groups in the entire cohort.

*Excessive alcohol consumption is defined as >28 g/day ethanol in men and >14 g/day in women.

AFP: alpha-fetoprotein, BMI: body mass index, DM: diabetes mellitus, GNRI: geriatric nutritional risk index, IQR: interquartile range, PT: prothrombin time, T.bil: total bilirubin
